# Inhibition of Cavin3 Degradation by the Human Parainfluenza Virus Type 2 V Protein Is Important for Efficient Viral Growth

**DOI:** 10.3389/fmicb.2020.00803

**Published:** 2020-04-30

**Authors:** Keisuke Ohta, Yusuke Matsumoto, Machiko Nishio

**Affiliations:** Department of Microbiology, School of Medicine, Wakayama Medical University, Wakayama, Japan

**Keywords:** human parainfluenza virus type 2, V protein, Cavin3, caveolae, lipid raft

## Abstract

Cavin proteins have important roles in the formation of caveolae in lipid raft microdomains. Pulse-chase experiments of cells infected with human parainfluenza virus type 2 (hPIV-2) showed decreased proteasomal degradation of Cavin3. Overexpression of hPIV-2 V protein alone was sufficient to inhibit Cavin3 degradation. Immunoprecipitation analysis revealed that V protein bound to Cavin3. Trp residues within C-terminal region of V protein, as well as the N-terminal region of Cavin3, are important for V–Cavin3 interaction. Cavin3 knockdown suppressed hPIV-2 growth without affecting its entry, replication, transcription, or translation. Higher amounts of Cavin3 were observed in V protein-overexpressing cells than in control cells in lipid raft microdomains. Our data collectively suggest that hPIV-2 V protein binds to and stabilizes Cavin3, which in turn facilitates assembly and budding of hPIV-2 in lipid raft microdomains.

## Introduction

**FIGURE 1:**
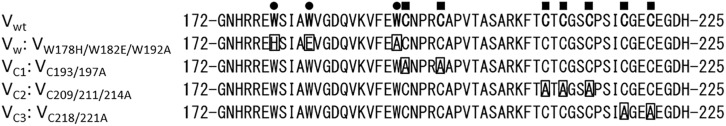
Amino acid sequences of the V-specific region of hPIV-2 V proteins. Trp and Cys residues are marked with filled circles and filled squares, respectively. Trp- and Cys-mutated V proteins used in this study are also shown.

Human parainfluenza virus type 2 (hPIV-2) is an enveloped, non-segmented, negative-strand RNA virus of the *Orthorubulavirus* genus of the *Paramyxoviridae*^1^ Its genome is composed of six tandem genes encoding the nucleocapsid (NP), phospho- (P), V, matrix (M), hemagglutinin-neuraminidase (HN), fusion (F), and large (L) proteins (Lamb and Parks, 2013). The unedited faithful copy of the P gene encodes the V open reading frame (ORF), while the edited insertion of two G nucleotides shifts the mRNA to the P ORF at the editing site (Ohgimoto et al., 1990). Thus, N-terminal amino acid sequences of P and V proteins are in common. Although V protein is not essential for hPIV-2 replication, the growth of V-deficient hPIV-2 is remarkably decreased relative to wt hPIV-2 (Nishio et al., 2005; Ohta et al., 2016b) (Table 1). V protein has been found to interact with several host proteins, such as STATs (Nishio et al., 2005), AIP1/Alix (Nishio et al., 2007), TRAF6 (Kitagawa et al., 2013), tetherin (Ohta et al., 2016b), Graf1 (Ohta et al., 2016a), caspase1 (Ohta et al., 2018a), inactive RhoA (Ohta et al., 2018c), and profilin2 (Ohta et al., 2019). Most of these V partners interact with the C-terminal region of V protein containing three Trp and seven Cys residues that form a bowl-shaped depression (Figure 1) (Li et al., 2006). The one exception is Graf1 whose interaction site is the N-terminal V/P common region (Ohta et al., 2016a).

**TABLE 1:** Summary of the properties of wt hPIV-2 and rPIV-2 carrying V mutants.

**Viruses**	**Relative viral growth in HeLa cells**	**IFN signaling block**	**STAT degradation**	**IL-lβ inhibition**	**F-actin formation**
wthPIV-2	+++	+	+	+	+
rPIV-2/V_w_	+	–	–	–	–
rPIV-2/V_C1_	+	–	–	Not tested	Not tested
rPIV-2/V_C2_	+	–	–	–	Not tested
rPIV-2/V_C3_	+	–	Not tested	Not tested	Not tested
References	Nishio et al. (2005) and Ohta et al. (2016b)	Nishio et al. (2005)	Nishio et al. (2005)	Ohta et al. (2018a)	Ohta et al. (2018c)

Caveolae are non-clathrin-coated invaginations of the plasma membrane (Anderson, 1998). They regulate lipid homeostasis, membrane tension, trafficking, and endocytosis (Parton and del Pozo, 2013; Shvets et al., 2014). Caveolae are specialized lipid raft microdomains enriched in cholesterol and sphingolipids (Parton and Simons, 2007). Caveolin and cavin proteins are critical components of caveolae (Hansen and Nichols, 2010). Caveolins are caveolae coat membrane proteins with molecular weights of 20–24 kDa (Hansen and Nichols, 2010). Among three caveolin family members (Caveolin1–3), Caveolin1 and Caveolin3 are essential for caveolae formation in non-muscle cells and muscle cells, respectively (Drab et al., 2001; Galbiati et al., 2001), while Caveolin2 is dispensable (Razani et al., 2002). Cavin proteins interact with Caveolin1 to regulate caveolae formation and function (Hill et al., 2008). Cavin proteins are key cytoplasmic components of caveolae with molecular weights of 31–47 kDa (Hansen and Nichols, 2010). All cavin proteins (Cavin1–4) contain leucine zippers (LZs), PEST domains (enriched in Pro, Glu, Ser, and Thr residues), and phosphatidylserine binding sites. Cavin1 is recruited to the plasma membrane by caveolins, and is also required for caveolae formation (Hill et al., 2008; Liu and Pilch, 2008). Cavin2 promotes Cavin1 recruitment to caveolae and induces caveolae curvature (Hansen et al., 2009). Cavin3 is involved in the intracellular transport of caveolae (McMahon et al., 2009). Both Cavin2 and Cavin3 can bind to Cavin1, suggesting that these cavin proteins coordinate with each other to regulate caveolae formation (Hansen et al., 2009; Mohan et al., 2015).

Caveolae are involved in several viral lifecycles. Simian virus 40 (SV40), human coronavirus 229E, and hepatitis B virus enter cells through caveolae-dependent endocytosis (Pelkmans et al., 2001; Nomura et al., 2004; Macovei et al., 2010). Caveolin1 has important roles in particle formation of several enveloped viruses. Parainfluenza virus type 5 (PIV-5), a member of the family *Paramyxoviridae*, facilitates its assembly and budding at the surface of the plasma membrane by the binding of its M protein with Caveolin1, resulting in the promotion of viral growth (Ravid et al., 2010). Although the respiratory syncytial virus (RSV) protein that interacts with Caveolin1 has not been identified, filaments of RSV colocalize with Caveolin1, suggesting the importance of Caveolin1 for RSV virion maturation (Brown et al., 2002). Caveolin1 also promotes growth of influenza A virus (IAV) via interaction between Caveolin1 and its M2 protein (Sun et al., 2010). Caveolin1 seems to recruit viral components to where these viruses assemble and bud. In contrast, the role of cavin proteins during viral infection is poorly understood.

In the present study, we investigate the role of cavin proteins in hPIV-2 infection. We examine whether hPIV-2 infection affects protein expression level of each cavin protein. Using immunoprecipitation, we analyze the interactions between cavin proteins and hPIV-2 proteins. We also examine the effects of cavin levels on hPIV-2 growth.

## Materials and Methods

### Cells and Viruses

Vero cells were grown in Eagle’s minimal essential medium (MEM) supplemented with 10% fetal calf serum (FCS). CV-1 Origin, SV40 (COS), HeLa cells, and their derivatives were grown in Dulbecco’s modified Eagle’s MEM (DMEM) containing 10% FCS. A HeLa cell line constitutively expressing wt V (HeLa/wt V) or Trp-mutated V protein (HeLa/V_W__178__H/W__182__E/W__192__A_) was previously described (Nishio et al., 2005). All cells were maintained in a humidified incubator at 37°C with 5% CO_2_. In this study, wt hPIV-2 (Toshiba strain) and rPIV-2/V_W__178__H/W__182__E/W__192__A_ (Nishio et al., 2005) were used.

### Antibodies and Reagents

A monoclonal antibody (mAb) against hPIV-2 V/P protein (315-1) was previously described (Nishio et al., 1997). Anti-FLAG mAb was obtained from Sigma (St. Louis, MO, United States). Anti-actin and anti-GAPDH mAbs were purchased from Wako (Osaka, Japan). Anti-Cavin3 polyclonal antibody (pAb) (SRBC Antibody: A302-418A) was obtained from Bethyl Laboratories (Montgomery, TX, United States). Anti-STAT2 pAb (C-20: sc-476) was obtained from Santa Cruz Biotechnology (Santa Cruz, CA, United States). Anti-Caveolin1 pAb and anti-Clathrin Heavy Chain mAb were purchased from Cell Signaling Technology (Danvers, MA, United States) and BioLegend (San Diego, CA, United States), respectively. MG132 was purchased from Wako.

### Plasmids

hPIV-2 V and P genes and their mutants were cloned into pcDL-SRα296 (Nishio et al., 1996, 1997). cDNA of Cavin3 was obtained from A549 cell total RNA by reverse-transcription (RT)-PCR as previously described (Ohta et al., 2018b). The cDNA and their deletion mutants were cloned into a pCMV-3Tag-8 vector with 3x FLAG tag at their C-termini (Stratagene, La Jolla, CA, United States). These constructs were all confirmed by DNA sequencing.

### Establishment of Cavin3 Knockdown Cell Line

DNA fragment encoding anti-Cavin3 short hairpin RNA (shRNA) was cloned into a pHygH1dTO (Takei et al., 2006). The shRNA target sequence of Cavin3 was 5′-GCACCGGATTGCAGAAGGT-3′ (corresponding to nucleotides 539–557 of the Cavin3 gene). HeLa cells were transfected with pHygH1dTO carrying anti-Cavin3 shRNA using XtremeGENE HP (Roche, Basel, Switzerland) according to the manufacturer’s instructions. Stable transfectants were selected with 100 μg/ml hygromycin (Invitrogen). Clones showing highly efficient Cavin3 depletion were used as Cavin3 knockdown cell lines (HeLa/Cavin3 KD).

### Quantitative Real-Time RT-PCR (qRT-PCR)

Total RNAs were isolated from HeLa cells using Isogen (Nippon Gene, Tokyo). cDNA synthesis was performed using a PrimeScript RT reagent kit (Takara, Kyoto, Japan) with oligo-dT_12__–__18_. qRT-PCR was carried out using Brilliant III Ultra-Fast SYBR Green QPCR Master Mix (Agilent Technologies, Santa Clara, CA, United States). The primers used were (5′–3′): Cavin3, forward, TCCAGAAGGCACCAGAGC, and reverse, CTGTACC TTCTGCAATCCGGT; the glyceraldehyde-3-phosphate dehy- drogenase (GAPDH) (used as an internal control), forward, GAAGGTCGGAGTCAACGGATTT, and reverse, ATCTTGA GGCTGTTGTCATACTTCT. The primers used for amplifying hPIV-2 genome, antigenome, and mRNAs were previously described (Matsumoto et al., 2016). Copy numbers of hPIV-2 genome, antigenome, and mRNAs were measured by qRT-PCR as previously described (Matsumoto et al., 2016).

### Immunoblot and Immunoprecipitation Assays

COS cells in 12-well plates were transfected with plasmids encoding Cavin3-FLAG or its mutants together with hPIV-2 V or P protein using XtremeGENE HP. At 2 days post-transfection, cells were harvested and sonicated for 30 s three times in lysis buffer containing 50 mM Tri-HCl (pH 7.4), 150 mM NaCl, and 0.6% NP-40. After centrifugation, supernatants were separated by SDS-PAGE, transferred to a nitrocellulose membrane, and analyzed by Western blotting (WB). For immunoprecipitation, the supernatants were incubated with nProtein A Sepharose 4 Fast Flow (GE Healthcare Bio-Sciences, Piscataway, NJ, United States) preincubated with anti-V/P or FLAG mAb. Precipitated proteins were analyzed by WB.

### Pulse-Chase Experiments of Cavin3

HeLa cells grown in 12-well plates were incubated in DMEM without methionine/cysteine for 1 h, and then labeled with DMEM containing 20 μCi/mL of [^35^S]methionine/cysteine (Perkin-Elmer, Boston, MA, United States) for 2 h. After being washed with normal DMEM, cells were incubated for various times. The cell lysates were subject to immunoprecipitation using anti-Cavin3 pAb or anti-GAPDH mAb, followed by SDS-PAGE.

### Plaque Assay

Vero cells grown in 12-well plates were infected with hPIV-2 diluted serially 10-fold in MEM without FCS, and cultured in MEM containing 2% FCS and 1.6% SeaKem ME agarose (FMC BioProducts, Rockland, ME, United States). The cells were stained with 0.05% neutral red at 5 days post-infection (dpi), and the number of plaques was counted.

### Isolation of Detergent-Insoluble Membrane Domain

HeLa cells grown in 12-well plates were incubated in ice-cold lysis buffer containing 50 mM Tri-HCl pH 7.4, 150 mM NaCl, and 1% TritonX-100. Supernatants were centrifuged and collected as soluble fractions. The remaining cells were lysed in ice-cold RIPA buffer containing 50 mM Tri-HCl pH 7.4, 150 mM NaCl, 1% NP-40, 0.1% SDS, and 0.5% sodium deoxycholate. After centrifugation, supernatants were collected as insoluble fractions.

## Results

### hPIV-2 Infection Inhibits Proteasome-Dependent Degradation of Cavin3

**FIGURE 2:**
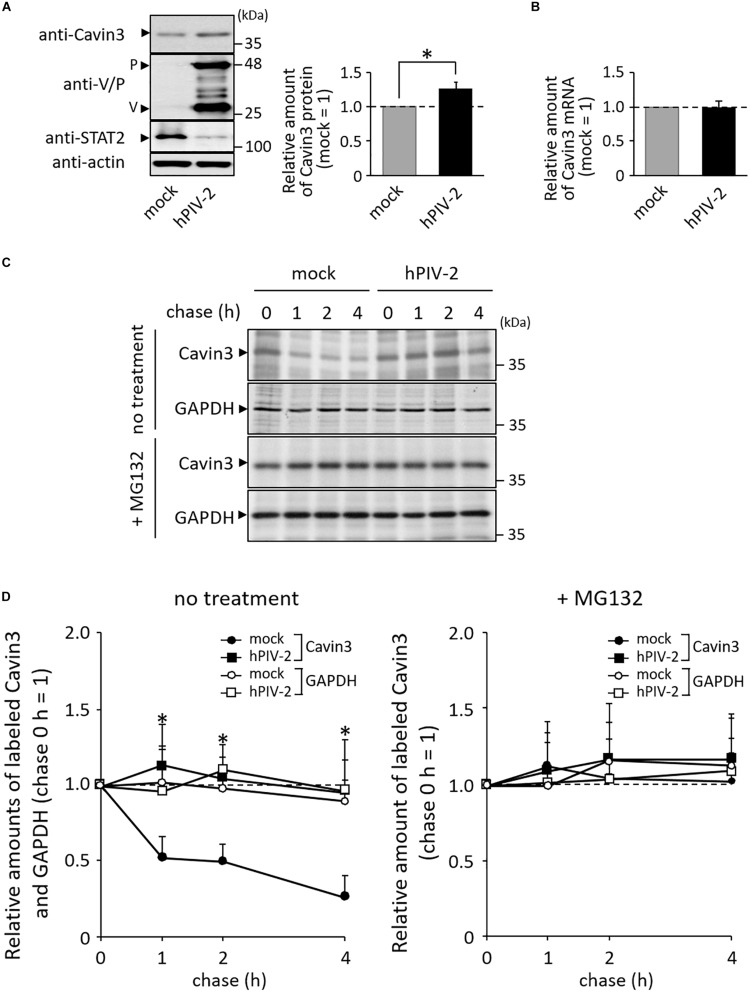
Effects of hPIV-2 infection on Cavin3 degradation. **(A)** HeLa cells were infected with or without hPIV-2 at an MOI of 1 for 1 day, and the cell lysates were subjected to immunoblot using the indicated Abs. Actin was used as a loading control. Bars show the quantitative densitometry of Cavin3 using ImageJ software (http://rsb.info.nih.gov/ij). The data are the means from three independent experiments, and are shown as the relative value (mock = 1). *P < 0.05, compared to values of mock. Error bars indicate standard deviations. **(B)** HeLa cells were infected with hPIV-2 under the same conditions as in **(A)**, total RNA was extracted, and the Cavin3 mRNA level was measured using qRT-PCR. The Cavin3 mRNA level was normalized to GAPDH mRNA expression. The data are the means from three independent experiments, and are presented as the relative values (mock = 1). Error bars indicate standard deviations. **(C)** HeLa cells were infected with hPIV-2 under the same conditions as in **(A)**, and then labeled with [^35^S]methionine/cysteine (20 μCi/mL) for 2 h. After removal of labeled methionine/cysteine, cells were chased in normal DMEM for the indicated times (top panel). The experiments were also performed in the presence of 0.1 μg/mL MG132 during pulse and chase periods (third panel). The cell lysates were analyzed by immunoprecipitation using anti-Cavin3 pAb or anti-GAPDH mAb and SDS-PAGE. GAPDH was used as a control (second and bottom panel). **(D)** The quantitative densitometry of Cavin3 and GAPDH in **(C)** was performed as described in **(A)**. The data are the means from more than three independent experiments, and are shown as the relative value (chase 0 h = 1). *P < 0.05, compared to values of Cavin3 amounts in mock cells. Error bars indicate standard deviations.

We investigated the effects of hPIV-2 infection on cavin protein expression levels. hPIV-2 infection appeared to cause a slight increase in Cavin3 protein levels in contrast to decrease in STAT2 protein levels (Figure 2A). We examined whether Cavin3 expression increases at the level of transcripts, but the mRNA level was not affected by hPIV-2 infection (Figure 2B). Cavin3 possesses two PEST sequences (Izumi et al., 1997), suggesting its rapid degradation. We therefore performed pulse-chase experiments of Cavin3 in hPIV-2-infected cells. HeLa cells were infected with hPIV-2 at an MOI of 1 for 1 day (or mock-infected), followed by pulse-labeling with [^35^S]methionine/cysteine. After 1, 2, and 4 h incubation, a greater amount of labeled Cavin3 remained in wt hPIV-2-infected cells than in mock-infected cells (Figure 2C, upper panel and Figure 2D). Inhibition of proteasomal degradation by MG132 resulted in suppression of Cavin3 degradation in both mock-infected and hPIV-2-infected cells (Figure 2C, middle panel and Figure 2D). hPIV-2 infection apparently inhibits proteasome-dependent degradation of Cavin3.

### V Protein Inhibits Degradation of Cavin3

**FIGURE 3:**
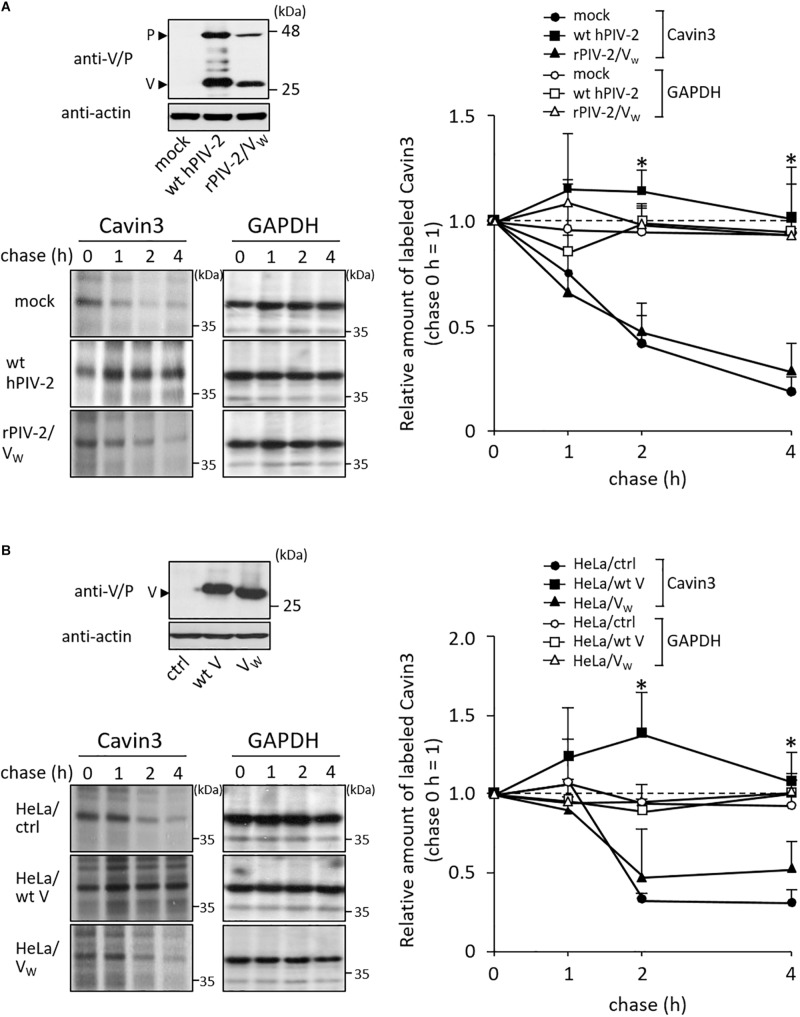
Effects of V protein on Cavin3 degradation. **(A)** HeLa cells were infected with wt hPIV-2 or rPIV-2/V_W__178__H/W__182__E/W__192__A_ (rPIV-2/V_W_), and the cell lysates were subjected to immunoblot using anti-V/P mAb (upper panel). Actin was used as a loading control. Pulse-chase experiments of Cavin3 and GAPDH in the infected cells were performed as shown in Figure 2C. The line graph shows the quantitative densitometry of Cavin3 and GAPDH performed as described in Figure 2D. GAPDH was used as a control. *P < 0.05, compared to values of mock. **(B)** The lysates of HeLa/ctrl, HeLa/wt V, and HeLa/V_W__178__H/W__182__E/W__192__A_ (HeLa/V_W_) were subjected to immunoblot using anti-V/P mAb (upper panel). Actin was used as a loading control. These cell lines were subjected to pulse-chase experiments as described in Figure 2C. The line graph shows the quantitative densitometry of Cavin3 and GAPDH performed as shown in Figure 2D. GAPDH was used as a control. *P < 0.05, compared to values of HeLa/ctrl. All experiments were performed at least three times independently.

We previously generated various recombinant hPIV-2s (rPIV-2s). These viruses lost several properties that wt hPIV-2 possesses (Table 1). HeLa cells were infected with one of these rPIV-2s, rPIV-2 carrying a Trp-mutated V protein (rPIV-2/V_W_: rPIV-2/V_W__178__H/W__182__E/W__192__A_) (Figures 1, 3A, upper panel). Cells were then pulsed with [^35^S]methionine/cysteine, and chased several times. Unlike wild type (wt) hPIV-2 infection, infection of rPIV-2/V_W__178__H/W__182__E/W__192__A_ did not affect the rate of Cavin3 degradation (Figure 3A).

To examine whether V protein can independently inhibit Cavin3 degradation, HeLa cells constitutively expressing wt V (HeLa/wt V) and Trp-mutated V protein (HeLa/V_W_: HeLa/V_W__178__H/W__182__E/W__192__A_) were used next (Figure 3B, upper panel). HeLa/V, HeLa/V_W__178__H/W__182__E/W__192__A_, and their control cells (HeLa/ctrl) were subjected to pulse-chase experiments. Overexpression of wt V reduced the rate of Cavin3 degradation at 2 and 4 h incubation (Figure 3B). Cavin3 degradation pattern in HeLa/V_W__178__H/W__182__E/W__192__A_ was similar to that in HeLa/ctrl (Figure 3B). These results indicate that V protein independently inhibits degradation of Cavin3.

### V Protein Binds to Cavin3

**FIGURE 4:**
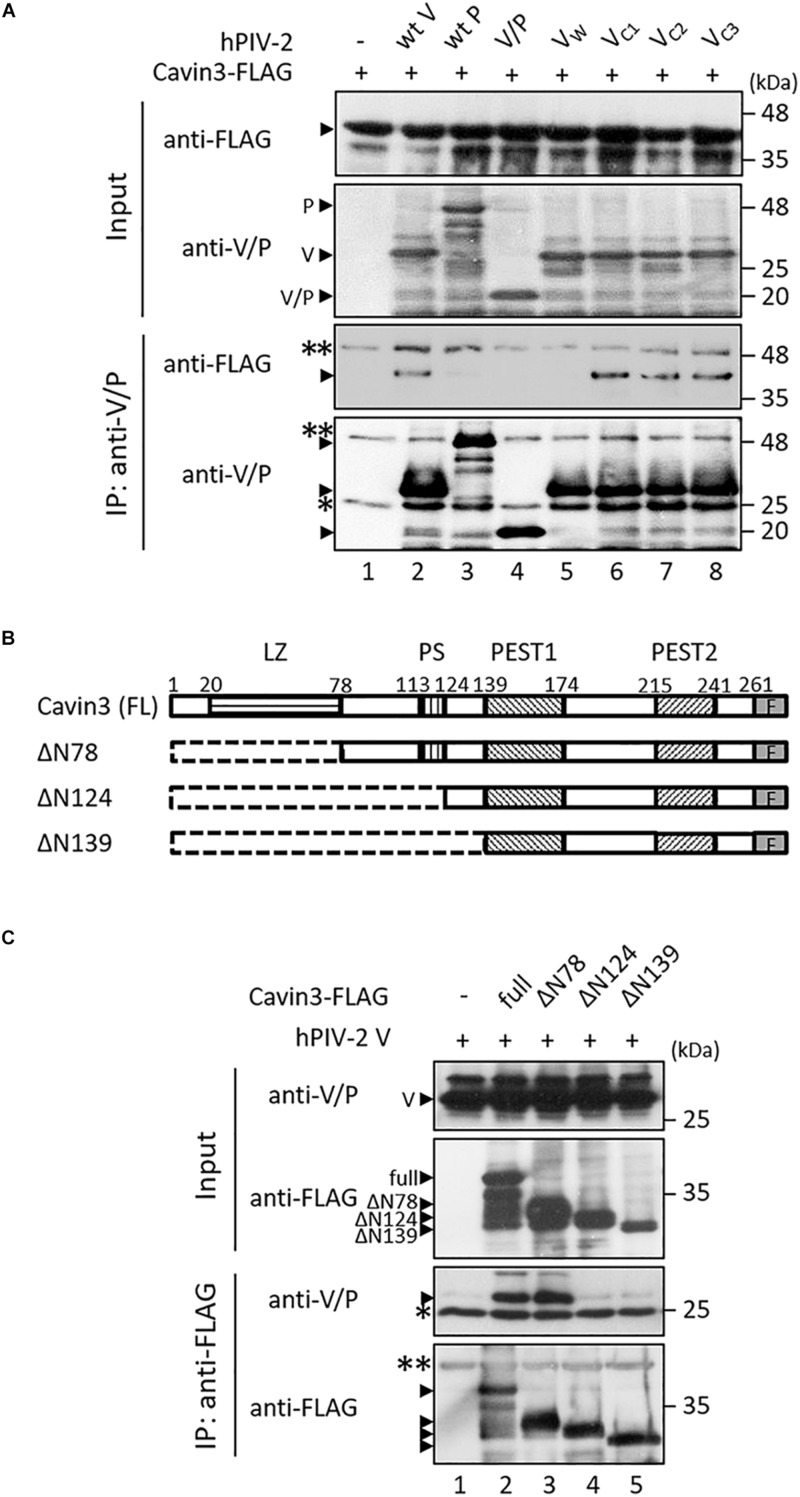
Interactions between Cavin3 and hPIV-2 proteins. **(A,C)** COS cells were transfected with various combinations of the indicated plasmids. V/P indicates a deletion mutant composed of only common regions of V and P proteins. V_W_, V_C__1_, V_C__2_, and V_C__3_ indicate V_W__178__H/W__182__E/W__192__A_, V_C__193__/__197__A_, V_C__209__/__211__/__214__A_, and V_C__218__/__221__A_, respectively. After 2 days, cell lysates were analyzed directly by immunoblotting (input). Immunoprecipitates with anti-V/P **(A)** or anti-FLAG mAb **(C)** were probed by anti-FLAG and anti-V/P mAbs. Double and single asterisks indicate immunoglobulin heavy chain and light chain, respectively. All experiments were performed at least three times independently. **(B)** Schematic diagram of full-length (FL) Cavin3 and its deletion mutants with C-terminal FLAG tag (F) was shown. Cavin3 contains leucine zipper (LZ), two PEST domains (PEST1 and PEST2), and phosphatidylserine-binding sites (PS). Deleted regions are indicated by the dotted lines.

We investigated the interaction between Cavin3 and hPIV-2 V and P proteins using immunoprecipitation. COS cells were transfected with SRα encoding hPIV-2 V or P gene together with FLAG-tagged Cavin3. Cavin3 was co-immunoprecipitated by V, but not P proteins (Figure 4A, lanes 1–3), indicating that the C-terminal region of V protein is important for the binding with Cavin3. As expected, a deletion mutant composed of only common regions of V and P proteins (V/P) could not bind to Cavin3 (Figure 4A, lane 4). There are three Trp and seven Cys residues in C-terminal V-specific region, which are important for interaction with several host proteins (Figure 1, and see “Introduction” section). To examine whether these residues are involved in binding with Cavin3, Trp-mutated V proteins (V_W_: V_W__178__H/W__182__E/W__192__A_) and Cys-mutated (V_C__1_: V_C__193__/__197__A_, V_C__2_: V_C__209__/__211__/__214__A_, and V_C__3_: V_C__218__/__221__A_) (Figure 1) were subjected to immunoprecipitation. Trp mutation lost the Cavin3 binding capacity, while all Cys mutants could bind to Cavin3 (Figure 4A, lanes 5–8). To identify the Cavin3 region important for V protein binding, three deletion mutants of Cavin3 with C-terminal FLAG tag were prepared (Figure 4B). Deletion of aa 1–78 of Cavin3 (ΔN78) did not affect V binding (Figure 4C, lane 3). In contrast, N-terminally deleted Cavin3 consisting of aa 125–261 (NΔ124) and aa 140–261 (NΔ139) could not bind to the V protein (Figure 4C, lanes 4 and 5). Thus, aa 79–124 of Cavin3 are important for the binding to V protein.

### Cavin3 Positively Regulates hPIV-2 Growth

**FIGURE 5:**
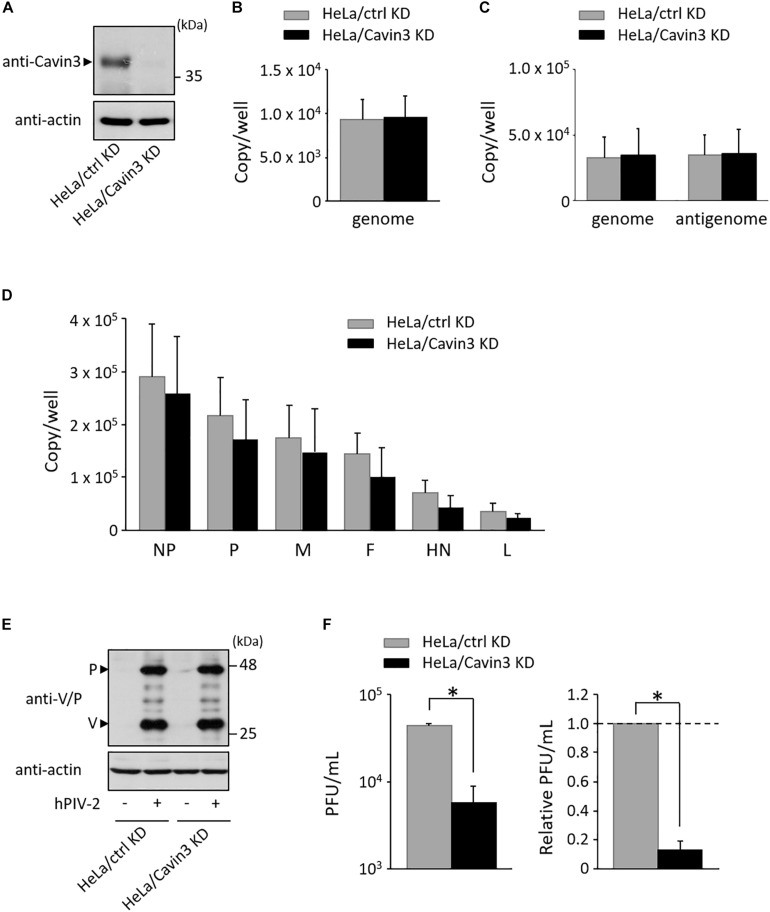
Effects of Cavin3 on hPIV-2 growth. **(A)** Lysates of the indicated cell lines were subjected to immunoblot using anti-Cavin3 pAb. Actin was used as a loading control. **(B)** HeLa/ctrl KD and HeLa/Cavin3 KD were incubated with hPIV-2 at an MOI of 1 for 60 min. The cells were then washed with PBS, and total RNA was extracted using Isogen. Copy numbers of hPIV-2 genome were measured by qRT-PCR. The data are the means from six independent experiments. Error bars indicate standard deviations. **(C,D)** HeLa/ctrl KD and HeLa/Cavin3 KD were infected with hPIV-2 at an MOI of 1 for 1 day. Total RNA was extracted and copy number of hPIV-2 genome and antigenome **(C)** or mRNAs **(D)** were measured by qRT-PCR. **(E,F)** HeLa/ctrl KD and HeLa/Cavin3 KD were infected with hPIV-2 under the same conditions as in **(C)**. The cell lysates were subjected to immunoblot using anti-V/P mAb **(E)**. Actin was used as a loading control. The amount of viruses in the culture supernatants was measured by plaque assay **(F)**. The values of PFU/mL are shown as the means from three independent experiments. Data are also shown as relative PFU/mL values (HeLa/ctrl KD = 1). *P < 0.05, compared to values of HeLa/ctrl KD. Error bars indicate standard deviations.

To investigate the effects of Cavin3 levels on hPIV-2 growth, a Cavin3 knockdown HeLa cell line (HeLa/Cavin3 KD) was generated (Figure 5A). To examine whether Cavin3 affects hPIV-2 entry, HeLa/Cavin3 KD and its control cell line (HeLa/ctrl KD) were incubated with hPIV-2 at an MOI of 1, and hPIV-2 genome in these cell lines was quantified by qRT-PCR. The amounts of hPIV-2 genome in HeLa/Cavin3 KD were similar to those in HeLa/ctrl KD (Figure 5B). To investigate the effects of Cavin3 on hPIV-2 replication, transcription, and protein synthesis, HeLa/Cavin3 KD and HeLa/ctrl KD were infected with hPIV-2 at an MOI of 1 for 1 day, followed by qRT-PCR and immunoblotting. Cavin3 knockdown did not affect hPIV-2 replication (Figure 5C), transcription (Figure 5D), or translation (Figure 5E).

We next examined whether Cavin3 is involved in virus production. HeLa/Cavin3 KD and HeLa/ctrl KD were infected with hPIV-2 at an MOI of 1 for 1 day, and the amount of viruses in the culture supernatants was measured by plaque assay. The virus production level in HeLa/Cavin3 KD was approximately 10-fold lower than that in HeLa/ctrl KD (Figure 5F). These results indicate that Cavin3 positively regulates hPIV-2 growth without affecting its entry, replication, transcription, or translation.

### V Protein Increases the Level of Cavin3 in Lipid Raft Microdomains

**FIGURE 6:**
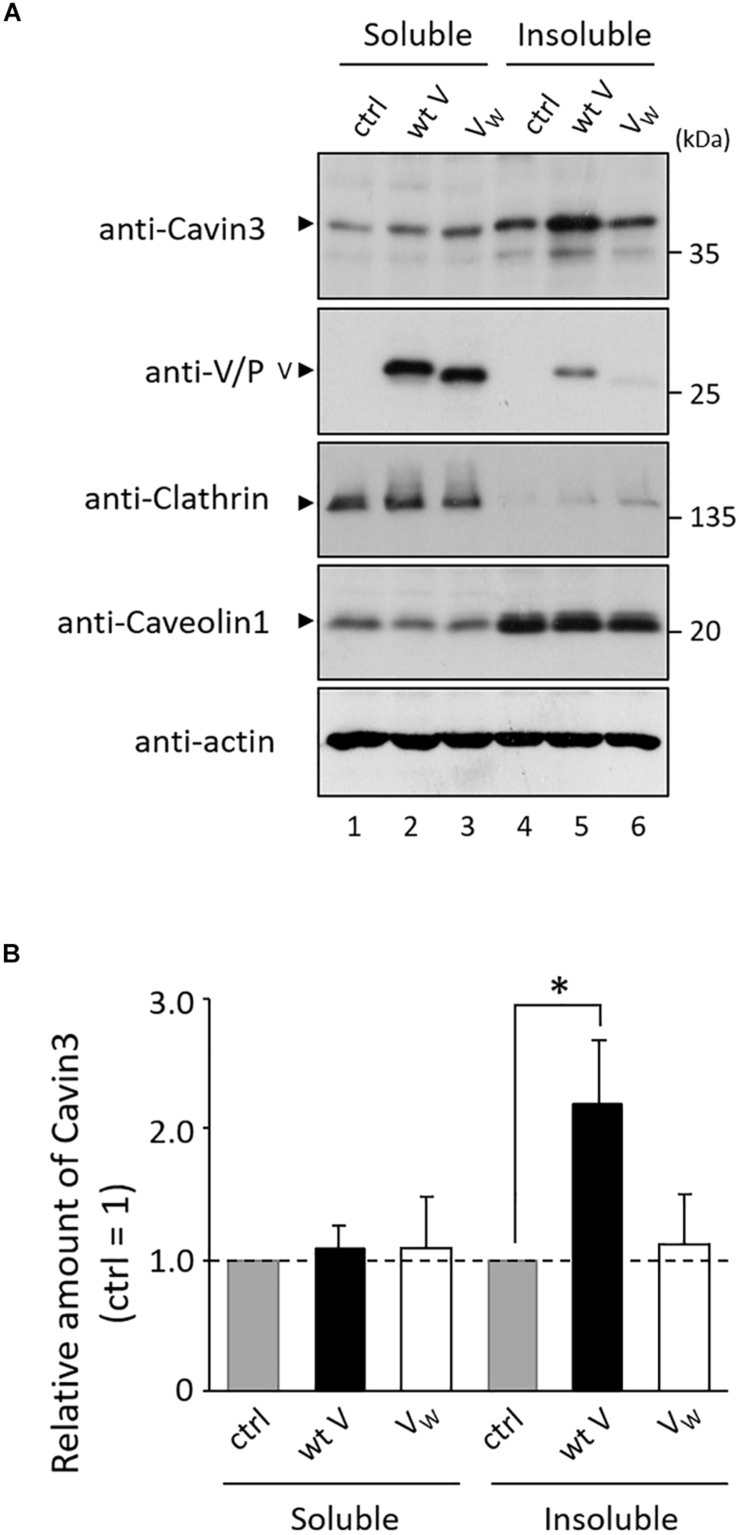
Effects of hPIV-2 infection on Cavin3 expression in lipid raft. **(A)** HeLa/ctrl, HeLa/wt V, and HeLa/V_W__178__H/W__182__E/W__192__A_ (HeLa/V_W_) were extracted with 1% TritonX-100 at 4°C, and soluble and insoluble fractions were prepared as described in the Materials and Methods section. Cells were subjected to immunoblot using the indicated Abs. Clathrin and Caveolin1 were used as non-raft and raft markers, respectively. Actin was used as a loading control. **(B)** The quantitative densitometry of Cavin3 was performed as described in Figure 2D. Data are shown as the relative value (HeLa/ctrl = 1). *P < 0.05, compared to values of HeLa/ctrl. Error bars indicate standard deviations. All experiments were performed three times independently.

Caveolae are a subpopulation of lipid rafts, where paramyxovirus budding occurs (Lamb and Parks, 2013). We investigated whether V protein is involved in the expression of Cavin3 in lipid rafts. Lipid raft microdomains can be defined as fractions that are insoluble in TritonX-100 at 4°C (Fiedler et al., 1993). HeLa/wt V, HeLa/V_W__178__H/W__182__E/W__192__A_ (HeLa/V_W_), and HeLa/ctrl were treated with lysis buffer containing 1% TritonX-100 at 4°C, and the amount of Cavin3 in detergent-insoluble fractions was quantified using immunoblotting. We confirmed the soluble/insoluble fractionation using Caveolin1 (a raft marker) and Clathrin (a non-raft marker) (Figure 6A). The expression levels of Cavin3 in the soluble fractions were not affected by wt V protein (Figure 6A, lanes 1–2). Insoluble fractions in HeLa/wt V contained significantly larger amounts of Cavin3 than those in HeLa/ctrl (Figure 6A, lanes 4–5 and Figure 6B). In contrast, the expression level of Cavin3 in HeLa/V_W__178__H/W__182__E/W__192__A_ was similar to that in HeLa/ctrl in both soluble and insoluble fractions (Figure 6A, lanes 1, 3, 4, and 6). These results suggest that hPIV-2 V protein increases Cavin3 levels in lipid raft microdomains.

## Discussion

We found that hPIV-2 V protein inhibits Cavin3 degradation (Figure 3), in contrast to its promotion of STAT2 degradation (Figure 2A) (Nishio et al., 2001, 2005). V protein seems to both positively and negatively regulate the stability of some host proteins. We expected that Cavin3 would be degraded through the calpain-dependent pathway because it possesses PEST sequences (Rogers et al., 1986; Shumway et al., 1999). Its degradation was inhibited, however, by MG132, a proteasome inhibitor (Figure 2C, middle panel). Cavin1 and Cavin2, both of which contain PEST sequences, are also degraded by the proteasome pathway (Breen et al., 2012; Tillu et al., 2015). PEST sequence is reported to be involved in proteasome-dependent degradation pathways (Spencer et al., 2004). However, as shown in Figure 4C, PEST sequences in Cavin3 were not the binding sites with V protein. V protein does not seem to directly mask the Cavin3 PEST sequences. Ubiquitylation of Lys residues within the phosphoinositide-binding site of Cavin1 leads to its degradation (Tillu et al., 2015). As aa 79–124 of Cavin3 was found to contain the V binding site (Figure 4C), V protein might mask the ubiquitylation sites within this region of Cavin3 by its binding.

Cavin3 levels positively contributed to hPIV-2 growth without affecting hPIV-2 entry, replication, transcription, or translation (Figures 5B–F). It is likely that a slight difference in the amounts of mRNA between HeLa/ctrl KD and HeLa/Cavin3 KD was negligible because the amounts of viral protein in HeLa/Cavin3 KD were similar to those in HeLa/ctrl KD (Figures 5D,E). Cavin3 level in lipid rafts (insoluble fractions) was increased by V protein (Figure 6A, lane 5). These results suggest the involvement of Cavin3 in the assembly and budding of hPIV-2 since lipid rafts are assembly and budding sites for several enveloped viruses, including measles virus (Vincent et al., 2000), Newcastle disease virus (NDV) (Laliberte et al., 2006), RSV (Brown et al., 2002), and IAV (Scheiffele et al., 1999; Takeda et al., 2003). Caveolin1 is incorporated in particles of PIV-5 (Ravid et al., 2010), NDV (Laliberte et al., 2006), and RSV (Brown et al., 2002). RSV particles also contain Cavin1 (Ludwig et al., 2017). However, Cavin3 was not observed in hPIV-2 virions (data not shown).

When detergent-insoluble fractions of PIV-5 infected cells were separated on sucrose gradients, its M protein fractionated in Caveolin1-rich fractions (Ravid et al., 2010), indicating that this M protein interacts with Caveolin1 in lipid raft microdomains. Caveolin1 binds to aromatic amino acid-rich region (FXXXXWXXF, corresponding to aa 355–363) of PIV-5 M (Ravid et al., 2010). As this region is conserved in hPIV-2 M protein, hPIV-2 M protein seems to be clustered by Caveolin1. A faint amount of V protein was detected in detergent-insoluble fractions (Figure 6A, lane 5). V protein might interact with Cavin3 before the recruitment of Cavin3 to the lipid rafts. The V protein in detergent-insoluble fractions (Figure 6A, lane 5) may have been recruited by Cavin3. The role of V–Cavin3 interaction appears to be quite different from that between Caveolin1 and the viral proteins. It is reported that Cavin3 interacts with Cavin1 and Caveolin1, which leads to an increase in surface dynamics of caveolae (Mohan et al., 2015). hPIV-2 V protein might enhance the Cavin3-induced activation of caveolae dynamics by stabilizing Cavin3 expression.

Our results collectively suggest that the hPIV-2 V protein binds and stabilizes Cavin3, which might activate the surface dynamics of caveolae that, in turn, increases the assembly and budding sites of hPIV-2, thus promoting hPIV-2 growth.

## Data Availability Statement

All datasets generated for this study are included in the article.

## Author Contributions

KO and MN designed the study. KO and YM performed the experiments. KO drafted the manuscript. KO and MN revised the manuscript. MN supervised the experimental work. All authors have read, commented on, and approved the manuscript.

## Conflict of Interest

The authors declare that the research was conducted in the absence of any commercial or financial relationships that could be construed as a potential conflict of interest.

## Footnotes

1 https://talk.ictvonline.org/taxonomy
